# Translational control by eIF2α phosphorylation and its downstream effectors in AD brains and in primary microglia exposed to Aβ or tau oligomers

**DOI:** 10.1002/alz70855_098364

**Published:** 2025-12-23

**Authors:** Danielle Cozachenco, Ricardo A S Lima‐Filho, Mariana Chauvet, Gustavo Trevizani Stelzer, Juliana T S Fortuna, Luana Heimfarth, Fernanda Guarino De Felice, Ottavio Arancio, Mychael V. Lourenco, Sergio T. Ferreira

**Affiliations:** ^1^ Federal University of Rio de Janeiro, Rio de Janeiro, Rio de Janeiro, Brazil; ^2^ Queen's University, Kingston, ON, Canada; ^3^ D'Or Institute for Research and Education, Rio de Janeiro, RJ, Brazil; ^4^ Columbia University, New York, NY, USA

## Abstract

**Background:**

The major histopathological hallmarks of Alzheimer's disease (AD) are neurofibrillary tangles, composed of the tau protein, and fibrillar aggregates of Aβ peptide. Considerable evidence points to important roles for soluble Aβ oligomers (AβOs) and, more recently, tau oligomers (TauOs) in the pathogenesis of AD. We and others have demonstrated that Aβ and tau aggregates impair neuronal mRNA translation (protein synthesis) through inhibitory phosphorylation of the eukaryotic initiation factor 2 α (eIF2α‐P), and that this leads to cognitive impairment in AD. Microglia play an essential role in innate immune response, and in clearance of neurotoxins from the brain. Aberrant microglial activation is thought to represent an important causative factor in AD pathogenesis. Even though several cellular events underlying AD pathophysiology are well established, little is known on the mechanisms underlying loss of microglial homeostasis. Specifically, the regulation of microglial protein synthesis in AD remains elusive.

**Methods:**

In this work, we analyzed the regulation of protein synthesis in *postmortem* AD brains, and then specifically in primary microglia cultures exposed to AβOs or TauOs.

**Results:**

eIF2α‐P is increased and associated with both CERAD and Braak stages in the AD prefrontal cortex. eiF2a‐P is further associated with CERAD, but not with Braak stages, in the AD hippocampus, suggesting that Aβ and tau aggregates differentially affect eIF2α‐P in distinct brain anatomical regions. In cortical primary microglia cultures, neither AβOs nor TauOs changed eIF2α‐P levels, its downstream effector ATF4, or global protein synthesis rates at 6 or 24 h post‐exposure. Despite the absence of changes in these mechanisms, AβOs induced an increase in the levels of fractalkine receptor CX3CR1, and a trend of increase in high mobility group box‐1 (HMGB1) protein at 24 h of treatment.

**Conclusions:**

Our findings indicate that eIF2α‐P and its downstream signaling are not changed in microglia exposed to AβOs or TauOs. Future work is warranted to explore other mechanisms underlying mRNA translation in AD microglia.

